# Non-infectious chemotherapy-associated acute toxicities during childhood acute lymphoblastic leukemia therapy

**DOI:** 10.12688/f1000research.10768.1

**Published:** 2017-04-07

**Authors:** Kjeld Schmiegelow, Klaus Müller, Signe Sloth Mogensen, Pernille Rudebeck Mogensen, Benjamin Ole Wolthers, Ulrik Kristoffer Stoltze, Ruta Tuckuviene, Thomas Frandsen

**Affiliations:** 1 Department of Pediatrics and Adolescent Medicine, University Hospital Rigshospitalet, Copenhagen, Denmark; 2 Institute of Clinical Medicine, Faculty of Medicine, University of Copenhagen, Copenhagen, Denmark; 3 Department of Diabetes and Metabolism, University Hospital Rigshospitalet, Copenhagen, Denmark; 4 Department of Pediatrics, Aalborg University Hospital, Aalborg, Denmark

**Keywords:** acute lymphoblastic leukaemia, ALL, chemotherapy, side effects, toxicities

## Abstract

During chemotherapy for childhood acute lymphoblastic leukemia, all organs can be affected by severe acute side effects, the most common being opportunistic infections, mucositis, central or peripheral neuropathy (or both), bone toxicities (including osteonecrosis), thromboembolism, sinusoidal obstruction syndrome, endocrinopathies (especially steroid-induced adrenal insufficiency and hyperglycemia), high-dose methotrexate-induced nephrotoxicity, asparaginase-associated hypersensitivity, pancreatitis, and hyperlipidemia. Few of the non-infectious acute toxicities are associated with clinically useful risk factors, and across study groups there has been wide diversity in toxicity definitions, capture strategies, and reporting, thus hampering meaningful comparisons of toxicity incidences for different leukemia protocols. Since treatment of acute lymphoblastic leukemia now yields 5-year overall survival rates above 90%, there is a need for strategies for assessing the burden of toxicities in the overall evaluation of anti-leukemic therapy programs.

## Introduction

The best contemporary chemotherapy for childhood acute lymphoblastic leukemia (ALL) now yields 5-year overall survival (OS) rates above 90%, which reflects intensified chemotherapy with treatment stratification directed by the somatic mutations and early response to chemotherapy, better use of conventional anti-leukemic agents, and improved supportive care, including broad-spectrum antibiotics to combat opportunistic infections ^1, 2^. However, a significant proportion of leukemic deaths, not least for lower-risk patients, are caused by therapy rather than by the leukemia itself, and this is just the tip of the toxicity iceberg ^3^. Nearly all patients encounter mucositis and serious, though manageable, infections, and although various other severe, acute toxicities individually have relatively low incidences, almost 50% of all patients will be affected by at least one of these ^4^. Whereas recent high-throughput, cost-effective technologies have revolutionized our insight into the somatic mutational landscape of ALL, disease pathogenesis, and drug resistance mechanisms ^5^, our understanding of non-infectious chemotherapy-associated acute toxicities remains limited, including how to prevent and treat them. This reflects their rarity (calling for international collaboration), diverse definitions and capture strategies across study groups, lack of tissue specimens to map pathogenesis, and uncertain associations with common germline DNA variants ^6, 7^. This review summarizes recent advancements in the exploration of non-infectious, chemotherapy-associated acute toxicities and outlines strategies for future research.

## The toxicity scenario

Every organ can be affected by acute side effects of anti-leukemic chemotherapy, the most common being opportunistic infections, mucositis, central or peripheral neuropathy (or both), bone toxicities (including osteonecrosis, ON), thromboembolism (TE), sinusoidal obstruction syndrome (SOS), endocrinopathies (especially corticosteroid-induced adrenal insufficiency and hyperglycemia), high-dose methotrexate (HD-MTX)-induced nephrotoxicity, asparaginase-associated hypersensitivity, pancreatitis, and hyperlipidemia. Other toxicities, including myopathy and some rare inflammatory toxicities (for example, epidermolysis), will not be addressed in this review.

Few of the non-infectious acute toxicities are associated with clinically useful risk factors, and comparison of their frequency across various anti-leukemic treatment programs has been hampered by wide diversities in toxicity definitions, capture strategies, and reporting, thus hampering meaningful comparisons of toxicity incidences. The toxicities have traditionally been defined and graded according to the US National Cancer Institute Common Terminology Criteria for Adverse Events (CTCAE) ^8^. However, these are generic in their grading and frequently inappropriate for children ^9^ and for the acute toxicities seen during childhood ALL therapy. Accordingly, 15 international childhood ALL study groups (Ponte di Legno Toxicity Working Group, or PTWG) have developed consensus definitions for 14 acute toxicities ^7^.

## Mucositis

Mucositis is a debilitating adverse effect that is reported to occur in at least 40% of patients after high-dose anti-metabolites or DNA-damaging drugs, including high-dose alkylating agents given as part of conditioning therapy prior to hematopoietic stem cell transplantation (hSCT) ^10– 13^.

Risk factors for mucositis include low body weight, reduced renal function, low neutrophil counts, and elevated pre-therapeutic levels of inflammatory mediators ^10, 12, 14, 15^. In addition, the risk of severe mucositis has, albeit with conflicting results, been associated with common DNA polymorphisms, including the folate pathway methylenetetrahydrofolate reductase ( *MTHFR*, particularly C677T) ^16^ and DNA repair ^17^.

Oral mucositis ranges from soreness with erythema and edema to painful, ulcerative mucositis requiring narcotic analgesics, which may lead to poor nutritional status ^18^. Intestinal mucositis typically develops in parallel with abdominal pain, diarrhea or constipation, nausea, and vomiting, but oral and intestinal mucositis may not coincide ^18^. They both tend to peak at the time of neutrophil nadir 10 to 14 days after chemotherapy and typically resolve during the subsequent 5 to 10 days.

Gastrointestinal mucositis reflects release of damage-associated molecular patterns that are sensed by pattern recognition receptors such as Toll like-receptors, causing release of inflammatory cytokines propagating an inflammatory response ^19– 21^. This is followed by an ulceration phase and finally resolution ^19^. The normal intestinal microbiome may play a protective role by stimulating endothelial cell proliferation and mucous production, and intestinal dysbiosis due to chemotherapy and antibiotics could aggravate mucositis, but this awaits clinical validation ^21– 23^. Severe mucositis disrupts the intestinal immunological barrier and is a risk factor for systemic infections, although it has been most intensively studied in the hSCT setting ^22, 24^. Accordingly, intestinal mucositis defined by hypocitrullinemia ** reflecting a reduced population of functional enterocytes may be better than neutropenia at defining the risk period for bacteremia ^24^.

Although several studies have demonstrated temporal associations between gastrointestinal toxicity, systemic inflammation, and fever, infections can be proven in only less than 50% of febrile neutropenic episodes ^25, 26^, and the cause in microbiologically negative cases is more likely systemic inflammation—for example, C-reactive protein, interleukin-6, and *in vitro* cytokine production—than opportunistic microorganisms ^13, 27– 29^. This has led to the introduction of febrile mucositis as a complementary term to the ubiquitous febrile neutropenia ^28^. hSCT studies have linked systemic inflammation to adverse outcome and increased treatment-related mortality ^30, 31^. It is conceivable, but not yet shown, that this also holds true for ALL.

Numerous interventions have been tested for the prevention or amelioration of mucositis as reviewed and regularly updated by the Mucositis Study Group of the Multinational Association of Supportive Care in Cancer and International Society of Oral Oncology ^32^. Parenteral non-steroid anti-inflammatory drugs, anti-epileptics, neuroleptics, and opioids are still the mainstay of pain control, despite often being insufficiently effective ^33^. Probiotics containing lactobacillus species seem to reduce chemotherapy-induced diarrhea and mucositis but have been tested only in highly specific treatment settings and await formal testing in patients with chemotherapy-induced neutropenia and mucosa barrier dysfuntion ^34– 37^ (NCT02544685). Other less established interventions of some efficacy include intravenous glutamine, cryotherapy, recombinant keratinocyte growth factor-1, and low-level laser therapy for oral mucositis ^32^. However, most of these approaches have been studied only insufficiently (if at all) during ALL chemotherapy.

## Central neurotoxicity

Central nervous system (CNS) toxicities during treatment occur in 10% to 15% of patients with childhood ALL and cover a wide spectrum of syndromes with overlapping symptoms, including seizures ^38^, HD-MTX-related stroke-like syndrome (MTX-SLS) ^39^ with or without reduced consciousness, posterior reversible encephalopathy syndrome (PRES) ^40^, and steroid psychosis ^41, 42^, and these may result in permanent or progressive neurocognitive defects (for example, attention, executive function) ^43– 45^ with or without white matter changes on magnetic resonance imaging (MRI).

Corticosteroids frequently cause transient changes in sleep pattern, mood, and cognition, and this can be quite burdensome to both patients and parents ^46^. Corticosteroids may affect the neurotransmitters dopamine or serotonin, deregulate the hypothalamic-pituitary-adrenal (HPA) axis, and cause hippocampal injury ^47^. In general, the risk of acute, severe neurotoxicity cannot be predicted, but the risk is higher for children below six years and for treatment with dexamethasone compared with prednisolone, potentially reflecting higher CNS penetration and longer half-life in CNS of dexamethasone ^48– 50^. Germline DNA polymorphisms in genes related to drug disposition or neurogenesis or both have been associated with neurotoxicity ^51^, but these candidate gene associations remain to be validated.

Seizures occur in approximately 10% of children with ALL ^38^. They can occur both as an isolated symptom, together with various other CNS toxicities (for example, intracranial hemorrhage or thrombosis, PRES, or MTX-SLS), or second to electrolyte and metabolic disturbances or to infections. Many patients subsequently require long-term anti-convulsive therapy, female sex being a significant risk factor ^52^.

MTX-SLS, which is characterized by focal neurological deficits or hemiparesis and often accompanied by disturbances in speech, affect, or consciousness (or a combination of these), develops within two to three weeks (usually 2 to 14 days) after HD-MTX or intrathecal MTX administration and waxes and wanes over the subsequent hours to days and then resolves within a few days ^39, 53^. MTX interferes with the methionine/homocysteine pathway and purine *de novo* synthesis pathways, disrupts myelin, causes accumulation of homocysteine and adenosine, and influences neurotransmitter status with a strong excitatory effect on the *N*-methyl-D-aspartate receptor (NMDAR). Vitamin B _12_ deficiency can promote these disturbances ^54^. The incidence of SLS varies from less than 1% to 15% in the literature and appears to vary according to the scheduling and intensity of MTX and co-administration of other agents such as cyclophosphamide and Ara-C and appears more frequently in children older than 10 years ^39^. Most patients make a full recovery, although there are reports of persisting neurological deficits, and the risk of recurrence with subsequent MTX therapy is low ^39^. Dextromethorphan, a non-competitive antagonist to NMDAR, or aminophylline (more relevant for acute MTX-induced neurotoxicity) has been advocated on the basis of small series ^55, 56^. The effect may be dramatic, but the use of these interventions awaits formalized validation. MRI will not always be able to confirm MTX-SLS but often reveals characteristic changes allowing discrimination of MTX-SLS from PRES ^57^.

PRES is a clinico-radiological entity frequently seen during the first months of ALL therapy, reflecting disturbances of cerebrovascular autoregulation and inconsistently characterized by headache, altered mental status, seizures, and visual disturbances ^40, 58, 59^. It may have several causes, predominantly arterial hypertension, chemotherapy, and corticosteroids, but the exact cause can frequently not be determined in the individual patient ^58^. On cranial MRI, areas of vasogenic edema are predominant but not restricted to the posterior regions of the brain or being exclusively bilateral. Affected areas are hypointense on T1-weighted and hyperintense on T2-weighted MRI ^59^. In contrast to MTX-SLS, PRES is hyperintense on apparent diffusion-weighted coefficient MRI images.

Some patients develop frank psychosis during corticosteroid therapy ^41, 42^. There are no clear guidelines for their clinical management, but sleep medication and tranquilizers and, in severe cases, anti-psychotics (for example, risperidone) can be indicated ^60^.

Transverse myelitis is a very rare complication seen in children with or without hematological malignancies ^61^. It may occasionally be associated with malignant infiltration ^62^ but can also be seen as a result of intensive chemotherapy, and high-dose cytarabine, MTX, and vincristine have been suspected to play a role ^63^.

## Peripheral neuropathy

Peripheral motor or sensory neuropathy or both are common, usually caused by vincristine, and in general completely reversible but may require many months for improvement ^64, 65^. In severe cases, they are occasionally associated with Charcot-Marie-Tooth disease ^66, 67^.

Metabolic drug-drug interactions may enhance vincristine neurotoxicity ^68^. Vincristine is inactivated by the major drug-metabolizing CYP isoform in humans, CYP3A4, and the azoles ketoconazole, itraconazole, and posaconazole are potent inhibitors of CYP3A4 ^69^. The potency of the azoles fluconazole and voriconazole as CYP3A4 inhibitors are much lower but may be clinically significant at high doses. A few germline DNA variants and gene expression profiles have been associated with the risk of vincristine-induced neuropathy ^64, 70^.

## Bone toxicities

The pathophysiology of osteoporosis during ALL therapy is uncertain, but the leukemia itself and the use of corticosteroids may cause osteoporosis and fractures, including multifocal compression fractures of the spine ^71– 74^, and osteoporosis affects up to 20% of newly diagnosed children with ALL ^75^. Five-year cumulative incidence of fractures has been reported to be 10% to 15% with no overall incidence difference between post-induction prednisolone or dexamethasone, although for adolescents dexamethasone seems to be associated with a higher risk ^73, 76^.

The most severe skeletal complication is symptomatic ON, caused by bone death resulting from poor blood supply ^77^. The PTWG has published a consensus definition of ON that accounts for localization of ON, joint deformation and the impact of ON on symptoms and self-care ^7^. If routine MRI is performed, an even higher frequency of non-symptomatic ON will be detected ^78^. Thus, the overall reported frequency varies from less than 5% to more than 70%, and females and adolescents have the highest risk ^79, 80^. ON is mainly diagnosed during the second year of ALL therapy (that is, during maintenance therapy), although presentation can occur earlier or even after cessation of therapy ^78, 81^. Hips and knees are most commonly affected in both subclinical and clinical cases, and often multiple joints are involved ^77, 81^. Many will suffer from daily pain, decreased ability of physical activity (or even need of a wheelchair), and reduced quality of life ^81, 82^. ON can lead to joint articular surface collapse with debilitating arthritis and need for joint-preserving or joint replacement surgery during the early phase of ON or months or years later.

So far, the only proven preventive measure for ON is giving dexamethasone intermittently rather than continuously ^79^. Corticosteroids contribute to the development of ON through osseous lipocyte hypertrophy with resultant increased pressure within the bone, which can cause vascular collapse and necrosis, and corticosteroids can cause direct toxicity to osteocytes. Fat emboli, vasculitis, or microthromboemboli that cause vascular occlusion can also contribute. Accordingly, hyperlipidemia induced by corticosteroids and asparaginase has been suggested to be associated with increased risk of ON, although most studies have been inconclusive ^78, 83^.

Genetic risk factors have been reported in pathways associated with the glutamate receptor, bone, lipid and folate metabolism, thymidylate synthase, corticosteroid disposition, and adipogenesis, but the associations have in general not been validated ^78, 84– 86^.

The benefits of prognostication of ON by imaging await validation ^87, 88^. Future research should focus on potential risk factors for various grades and for single-versus-multiple site ON, on the association with metabolism of drugs that may influence lipid profiles and coagulation ^78^, on the long-term outcome of ON, on improved guidelines for treatment adaptation and interventive surgery, and on the association of germline DNA variants with phenotype subsets.

## Thromboembolisms

TE located to the venous system is most common, and half of the cases involve the CNS ^89– 91^. The cumulative incidence of symptomatic venous TE is 2 to 8% ^90– 93^, but asymptomatic cases have been reported in up to 70% of patients ^92, 94^. Risk factors for TE include the leukemia itself, older age, central line catheters, immobilization, infections, systemic inflammation, and therapy with asparaginase or corticosteroids or both ^76, 90, 93, 95, 96^, whereas inherited thrombophilia risk factors, including common germline DNA polymorphisms, do not seem to play a role or at best remain uncertain ^96^. The fatality rate of venous TE is highest in children with thromboses in cerebral veins, and studies on the benefits of anti-thrombotic prophylaxis, preferably with the novel oral anti-coagulants, are needed ^90, 97, 98^.

## Sinosoidal-obstruction syndrome

Until recently, SOS, previously known as veno-occlusive disease ^99^, has primarily been a serious complication of hSCT and is otherwise rare during childhood ALL therapy except with continuous oral thioguanine ^100^, not least in patients who carry low-activity alleles for thiopurin methyl transferase ^101^. Doppler ultrasound showing reversed hepatic portal flow may aid the diagnosis, but a normal flow does not exclude the diagnosis and thus is not a mandatory diagnostic requirement. Instead, at least three of five criteria need to be fulfilled: that is, hepatomegaly, hyperbilirubinemia above upper normal limit (UNL), ascites, weight gain at or above 5%, and thrombocytopenia (transfusion-resistant or otherwise unexplained by treatment or both) ^7^.

The pathogenesis remains unclear, but drug-induced damage to hepatic endothelium and microcirculation and subsequent ischemic hepatocellular necrosis are the presumed mechanism ^99, 102, 103^. Previously, SOS occurred extremely rarely during 6-MP therapy ^100^ but recently has been described as a frequent complication to continuous polyethylene glycol-linked *Escherichia coli* asparaginase preparation (PEG-asparaginase) during 6-MP-based maintenance therapy when combined with pulses of either HD-MTX or vincristine/dexamethasone, probably reflecting the impact of asparaginase on 6-MP pharmacokinetics causing higher drug metabolite levels ^104^. Management of SOS during thiopurine therapy follows the same principles as management of SOS following hSCT: that is, fluid and sodium chloride restriction, diuretics, and, in the rare severe cases, defibrotide.

## Endocrinopathies

There is a paucity of prospective longitudinal studies determining endocrine changes during ALL therapy, and the existing studies have small sample sizes. Growth retardation and relative growth hormone deficiency are common during ALL therapy, but usually an adequate growth catch-up is obtained after cessation of therapy in children who do not receive radiotherapy ^105, 106^, but with a trend toward reduced final height ^107^.

A significant weight gain is seen in up to 40% of children with ALL, primarily reflecting exposure to corticosteroids and reduced physical activity with insulin resistance, hyperglycemia, and prediabetes, which could indicate the need for dietary modifications and insulin therapy ^108– 111^. The risk of corticosteroid-induced hyperglycemia is aggravated by asparaginase therapy ^112, 113^. The prevalence of hyperglycemia during ALL therapy has been reported to be 10% to 20% during treatment with asparaginase and corticosteroids, most frequently in children above 10 years of age, with resolution after cessation or tapering down of these drugs ^112– 116^. Medication-induced diabetes may be a marker for metabolic disease later in life ^116^. Finally, hyperglycemia and obesity both have been associated with reduced event-free survival ^117, 118^.

Fasting hypoglycemia is common during MTX/thiopurine-based maintenance therapy, especially in children below 6 years of age, but resolves after discontinuation of therapy ^119, 120^. It may reflect lowered plasma levels of the gluconeogenic amino acids (alanine and glutamine) as well as impaired glycogenolysis or glyconeogenesis ^119, 121^.

Corticosteroids cause a suppression of the HPA axis with secondary adrenal insufficiency and impaired stress response in nearly all patients, which for some patients may last several months after cessation of corticosteroid therapy irrespective of whether prednisolone or dexamethasone has been used ^122^. It may be aggravated by co-administration of fluconazole ^122^. Thus, corticosteroid replacement is indicated during the first weeks to months after cessation of corticosteroid therapy, not least during episodes of serious stress unless a stimulation test has shown a normal adrenal response ^122, 123^. The duration of adrenal insufficiency has been ascribed to variants of the GR gene ^124^, but formal genome-wide association analyses are lacking.

## HD-MTX-related nephrotoxicity

Alkalinization and vigorous hydration reduce the risk of significant nephrotoxicity with HD-MTX, but approximately 3% of patients will experience severe renal toxicity that will further compromise MTX clearance ^125– 128^. The nephrotoxicity is likely to be related to precipitation of MTX crystals in the kidneys and this is partly due to insufficient hydration and alkalization ^129, 130^. Plasma creatinine usually peaks within a few days after initiation of the HD-MTX infusion and returns to baseline after a few weeks. Nearly all patients will subsequently tolerate full-dose HD-MTX without recurrent nephrotoxicity ^127, 128^. Higher doses of folinic acid, adjusted by the plasma MTX levels, are essential to limit the risk of life-threatening myelosuppression and mucositis, but whether over-rescue could increase the risk of relapse remains an unsolved challenge ^131– 133^. In cases with extremely delayed MTX clearance, glucarpidase may be helpful to degrade MTX by enzymatic cleavage to 2,4-diamino-N10-methyl-pteroic acid (DAMPA) and glutamate ^127, 128^, but it does not promote restoration of renal function. Proton pump inhibitors and non-steroidal anti-inflammatory drugs ^134– 142^ as well as foodstuff (for example, licorice ^143^) and beverages (with low pH or sweetened with licorice extract) have been suspected to affect the MTX clearance ^144^. Since the introduction of 5-HT3 receptor antagonists, emesis is not a problem during HD-MTX and not linked to acute kidney injury.

Trimethroprim-sulfamethoxazole used as *Pneumocystis jiroveci* prophylaxis during ALL therapy does not seem to interfere with HD-MTX PK ^145^.

Several germline DNA variants are associated with MTX clearance, most notably in *SLCO1B* ^146– 149^, but none has yet been implemented in HD-MTX dosing strategies or been shown to be associated with extremely delayed MTX clearance.

## Toxicities secondary to asparaginase therapy

Asparaginase causes a range of toxicities due to asparagine depletion and disturbed protein synthesis. These toxicities may occur in up to 20 to 25% of all patients ^4^ and may lead to discontinuation of asparaginase therapy, which may increase the risk of relapse, not least in the CNS ^150, 151^.

## Asparaginase-associated allergy

The various asparaginase preparations and recombinant analogs differ in their biologic half-lives (shortest for *Erwinia chrysanthemi*-derived asparaginase and longest for PEG-asparaginase) and in their immunogenicity (lowest for PEG-asparaginase) ^152, 153^. Asparaginase can induce antibody formation that neutralizes asparaginase with or without (so-called silent inactivation) clinical signs of hypersensitivity ^154– 156^. Identification of silent inactivation requires measurement of plasma asparaginase activity levels.

The reported frequency of allergic reactions ranges from 3 to 75% depending on the type, dose, route, and duration of asparaginase administration, and allergic reaction primarily occurs after the first or second dose and virtually always is associated with zero asparaginase activity ^150, 154, 157– 162^. The reactions range from mild, local reactions to life-threatening systemic responses, including urticaria, symptomatic bronchospasm, edema/angioedema, and hypotension. Premedication with corticosteroid and anti-histamines and increased infusion time can reduce allergic symptoms but do not prevent asparaginase inactivation, and thus symptoms of hypersensitivity indicate the need to switch from *E. coli*-derived preparations to Erwinia asparaginase (or vice versa) ^7, 163^. Less immunogenic asparaginase preparations are emerging but are not routinely used in first-line therapy ^164– 166^.

Allergic-like reactions (for example, vomiting, stomach ache, or rash) with intact asparaginase activity can be seen but do not indicate discontinuation of the drug ^7^. Therapeutic drug monitoring can be helpful for differentiating allergy and allergic-like reactions ^154^. HLA-DRB1*07:01 and genetic variations in *GRIA1* have been associated with a higher incidence of hypersensitivity and anti-asparaginase antibodies ^167, 168^.

## Asparaginase-associated pancreatitis

Asparaginase-associated pancreatitis (AAP) has a reported incidence of 2 to 18% depending on the cumulative asparaginase dose (that is, treatment duration) and toxicity capture strategies but seemingly not on the route of administration ^7, 158, 169– 174^. AAP is most often diagnosed within two weeks of asparaginase exposure (median of 11 days with PEG-asparaginase), but the interval may be longer ^175^. The diagnostic criteria defined by the PTWG ^7^ are similar to those developed for pancreatitis in general ^176^ and require two of three criteria to be met: (i) abdominal symptoms suggestive of AAP, (ii) characteristic findings of pancreatitis on imaging, and (iii) serum lipase or amylase or both at least three times the UNL, and both enzymes should be measured because of a poor correlation between the two ^175^. If imaging shows pancreatic necrosis or hemorrhage and/or the abdomimal symptoms and elevated pancreatic enzymes at least three times the UNL persist for more than 72 hours, AAP is classified as severe and otherwise as mild.

Most AAP episodes are accompanied by systemic inflammatory responses (fever, elevated heart rate, elevated respiratory rate, or hypotension) and thus may easily be misinterpreted as sepsis. In addition to transient or permanent discontinuation of asparaginase therapy, treatment of AAP includes appropriate triage, fluid resuscitation, antibiotics (until an infection is ruled out), and monitoring for and treatment of AAP-related complications ^177^. The mortality rate is low, but patients systemically affected at AAP diagnosis are at increased risk of developing pseudocysts, acute or persistent diabetes mellitus, and chronic/relapsing pancreatitis ^175, 178^. Octreotide has been tested in few patients, but the benefit thereof remains to be determined ^179, 180^.

The risk of a second AAP after re-exposing patients with AAP to asparaginase is almost 50% and does not seem to be significantly lower if the first AAP episode was classified as mild ^170, 174, 175^.

Risk factors for AAP are few, although the incidence is associated with older age. Polymorphisms in *PRSS1*, *SPINK1*, *ASNS*, *ULK2*, *RGS6*, and *CPA2* genes have been associated either with pediatric pancreatitis in general or with AAP ^162, 174, 181, 182^, although these associations await validation.

## Hyperlipidemia

Elevated triglycerides and cholesterol occur frequently during ALL therapy and are associated with corticosteroid and asparaginase therapy ^7, 78, 83, 183^. However, patients are generally completely unaffected, even when levels are 40 to 50 times the UNL, the association with specific toxicities is very uncertain, and accordingly neither routine measurements nor interventions are recommended ^7^.

The hypertriglyceridemia is likely related to an increase in the endogenous hepatic synthesis of very low-density lipoprotein combined with a decreased activity in lipoprotein lipase, an enzyme involved in the removal of triglyceride-rich lipoproteins from the plasma ^184^.

The most common preventive measures in cases of hypertriglyceridemia are dietary restrictions (very limited effect), fibrates, insulin infusions, heparin infusions, and in extreme cases plasmapheresis, but there are no data to support that any of these interventions reduces the risk of hypertriglyceridemia-associated toxicities ^83, 185– 189^. In adults with non-malignant disorders, hypertriglyceridemia (above 10 times the UNL) has been associated with an increased risk of acute pancreatitis ^185, 188, 190^, but so far this has not been replicated in children with ALL ^191^. A few studies have indicated associations with development of ON and thrombosis ^78, 83, 95, 158, 188, 192^, but no randomized studies have explored whether lipid-lowering interventions prevent these complications.

## Host genome variant associations

As mentioned above, multiple variants in germline DNA have been associated with the pharmacology of anti-leukemic agents, including the risk of toxicities ^6, 68, 193^, but their individual hazard ratios are generally low (<2.0), the variants are rare or lack validation in independent studies, and treatment alterations according to such variants so far have not been implemented in childhood ALL therapy. The main reasons for our current inability to identify clinically actionable germline variants associated with specific toxicities are lack of sufficient study power (since each toxicity is rare and few trial groups are investigating genotype variation), incomplete toxicity capture, lack of detailed phenotyping (for example, lumping all subtypes and grades of a toxicity), and exploration of single-nucleotide polymorphisms rather than biological pathways. To address these limitations, the PTWG is now collecting phenotypes of several acute toxicities (pancreatitis, ON, and CNS toxicities) in hundreds of patients for each of these toxicities to associate detailed phenotypes with germline DNA variants ^175^.

## Leukemia predisposition syndromes

Recent research has identified several germline mutations in genes that play a critical role in hematopoiesis and lymphoid development and that are also frequently somatically mutated in ALL, such as *PAX5* ^194, 195^, *ETV6* ^196, 197^, *RUNX11* ^198^, and *IKZF1* ^199^, which align with the findings of high subtype concordance in familial cases of ALL ^195, 197, 200, 201^. This indicates that pure familial ALL syndromes may constitute a substantial part of ALL etiology and that more such syndromes are expected to emerge in parallel with a growing number of patients being germline-sequenced and with a deeper understanding of the impact of coding and non-coding DNA interactions ^196^. However, the impact of such germline DNA mutations on toxicities, not least those involving the bone marrow and immune system, remains to be determined. The risk of second malignant neoplasms may also be increased when childhood ALL arises due to a predisposition syndrome. Unusual acute toxicities and second malignant neoplasms therefore should lead to clinical suspicion of an underlying syndrome ^202^.

Down syndrome is the most frequent known germline mutation predisposing to ALL and is associated with enhanced gastrointestinal toxicity ^203^. However, reducing treatment intensity may also increase the risk of relapse ^204^ and should be considered only in case of excessive toxicity in the 10 to 15% of Down syndrome-ALL patients who harbor high hyperdiploidy or an *ETV6-RUNX1* translocation, since these subsets have a superior cure rate ^205^.

Several other ALL-predisposing syndromes such as Li-Fraumeni, ataxia telangiectasia, Nijmegen breakage, biallelic mismatch repair, and Fanconi anemia can also exhibit syndrome-related toxicities when exposed to DNA-damaging anti-cancer agents or radiotherapy ^206– 209^. In such cases, a reduction of DNA-damaging drug doses must be considered on an individual basis, and at least for ataxia telangiectasia and Nijmegen breakage dose reduction may not be associated with an increased risk of relapse ^210^. In contrast, thiopurine-based maintenance therapy may be less efficient in patients with biallelic mismatch repair deficiency, since this pathway is critical for thiopurine cytotoxicity ^211^.

## Future research

The low frequency and poor definitions of most of the listed organ toxicities have hampered their in-depth exploration, including the impact of specific drug dosing regimens, and identification of clear risk factors for certain phenotypic subsets. The recent PTWG consensus definitions of 14 of these toxicities have provided a platform for international collaboration on these issues ^7^. The results from the first of such explorations demonstrate its feasibility ^175^ and may allow exploration of the association between risk factors, including host DNA variants, in well-defined phenotypic subsets and provide evidence-based guidelines for treatment adaptation. Furthermore, the association of these acute toxicities with the risk of long-term organ toxicities (for example, dementia, diabetes, arthrosis, and chronic pancreatitis) remains to be mapped. Currently, event-free survival measures encompass death during induction, resistance to first-line therapy, relapse of leukemia, non-leukemic death during clinical remission, and development of a second cancer. However, many patients with a late relapse or a second cancer have a fair chance of cure ^212, 213^, whereas chronic toxicities are generally irreversible and challenge patients’ ability to live a normal adult life ^214^. This calls for new endpoint measures that include both survival and quality of life, which will require common strategies for toxicity capture and registration, and international collaboration to identify host genome variants and exposures (for example, anti-leukemic treatment, co-medication, and diet) associated with the risk of specific toxicities, but it also demands the development of a joint endpoint scoring system that encompasses OS as well as severe toxicities, both acute and long-term.

## Abbreviations

6-MP, 6-mercaptopurine; AAP, asparaginase-associated pancreatitis; ALL, acute lymphoblastic leukemia; CNS, central nervous system; HD, high dose; HPA, hypothalamic-pituitary-adrenal; hSCT, hematopoietic stem cell transplantation; MRI, magnetic resonance imaging; MTX, methotrexate; NMDAR, N-methyl-D-aspartate receptor ON, osteonecrosis; OS, overall survival; PEG, polyethylene glycol; PRES, posterior reversible encephalopathy syndrome; PTWG, Ponte di Legno Toxicity Working Group; SLS, stroke-like syndrome; SOS, sinusoidal obstruction syndrome; TE, thromboembolism; UNL, upper normal limit.
